# Need of entomological criteria to assess zero transmission of gambiense HAT

**DOI:** 10.1371/journal.pntd.0009235

**Published:** 2021-03-25

**Authors:** Philippe Solano

**Affiliations:** Institut de Recherche pour le Développement, UMR INTERTRYP IRD-CIRAD, Université de Montpellier, Montpellier, France; Imperial College London, Faculty of Medicine, School of Public Health, UNITED KINGDOM

## Background

Human African trypanosomiasis, also known as sleeping sickness, is a neglected tropical disease due to trypanosomes (Kinetoplastidae) transmitted to humans by a vector, the tsetse fly (Diptera: Glossinidae), that only occurs in Africa. There are 2 forms of the disease: Rhodesiense HAT (r-HAT) is due to *Trypanosoma brucei rhodesiense* and occurs East and South of Africa. Gambiense HAT (g-HAT) is due to *Trypanosoma brucei gambiense*, occurs in West and Centre of Africa, and is considered as the chronic form of the disease. Only g-HAT will be considered in this paper, since it is the only one that is targeted for “elimination as interruption of transmission” by WHO in the 2030 WHO roadmap for NTDs.

The disease is lethal without treatment with some exceptions, and there is no vaccine. Control efforts have been usually implemented through diagnosis and treatment. The diagnosis involves a complex chain composed of clinical examination, a serological test, followed by a parasitological confirmation, and treatment is usually given only to people in whom trypanosomes have been detected. A diagnosis of the neurological stage still relies on lumbar puncture. Treatment has for long been based on arsenical derivatives showing severe side effects, then has improved through the NECT (Nifurtimox-Eflornithine combination therapy; [1]), and even better recently with the arrival of an oral treatment that can treat the 2 stages, the fexinidazole [2,3]. Combining medical activities with tsetse control in g-HAT foci can accelerate HAT control, as observed in Guinea [4], Chad [5], and D. R. Congo [6]. In 2018, less than 1,000 cases of HAT were reported [7], thanks to this combination of medical and vector control tools. It can be estimated that, currently, this has allowed decreasing and preventing HAT transmission in an area of more than 7,000 km^2^, protecting more than 1 million people living in these foci of 5 countries, Guinea, Cote d’Ivoire, Chad, DRC, and Uganda [8].

With this spectacular decrease in number of cases observed, some countries are in the process of submitting their dossier to WHO for the validation of elimination as public health problem, and some even are in the process of interrupting transmission. Within the context of “elimination as interruption of transmission,” a key question now becomes: what criteria should be used to assess this interruption of transmission? Here we will review how elimination efforts are nowadays being followed up in g-HAT, how this compares to other vector-borne NTDs (VB-NTDs), and we will review the entomological tools available that may be used for interruption of transmission.

## How is monitoring of elimination efforts followed up?

In endemic countries for g-HAT, there is usually a national control program for HAT within the Ministry of health. This program organises the activities at the central and peripheral levels, sometimes with international partners, and has indicators such as an annual number of cases, of seropositives, and of people screened for HAT, usually with a mapping of these indicators in the WHO atlas of HAT [9].

An important problem with indicators that only refer to medical activities is that they are intrinsically blind to nonhuman transmission. *T*. *b*. *gambiense* also circulates outside the human component, i.e., in the tsetse vector (males and females) that carry them throughout their life span, and in mammalian hosts that may constitute reservoirs, although the role of these reservoirs in g-HAT remains controversial (see [10] for recent review).

There is also a need to remind that g-HAT is a chronic disease where an infected subject can live years with his trypanosomes before showing signs of disease [11]. In addition, mobility of people including HAT patients within and outside g-HAT foci is not controllable, thereby reinforcing the need to have these local entomological parameters.

## What is being done in other VB-NTDs?

In Lymphatic Filariasis (LF), Transmission Assessment Surveys (TAS) are being implemented as a decision tool to know when mass drug administration (MDA) can be stopped [12]. These TAS consist of a screening of filarial infection in humans. More precisely, “the protocol aims at screening young children who were born after the mass treatment for filarial infection. If the number of infected children is smaller than the pre-defined number, mass treatment can be stopped. The same protocol is followed for periodical assessment to verify whether there are any new infections” [13]. An alternative to the TAS has been developed in the vector using xenomonitoring, i.e., detecting filarial DNA in the mosquito vector, with an infection threshold below which transmission is considered to be interrupted.

In Onchocerciasis, a combination of tests is used to confirm the interruption of transmission of *Onchocerca volvulus*. These tests consist in a combination of an O-150 PCR detecting DNA of *O*. *volvulus* in black flies and an Ov-16 serological test in children, followed by PCR on skin snips for those positives at the serological test [14].

## In g-HAT, what are the entomological tools used?

**Fig 1:**
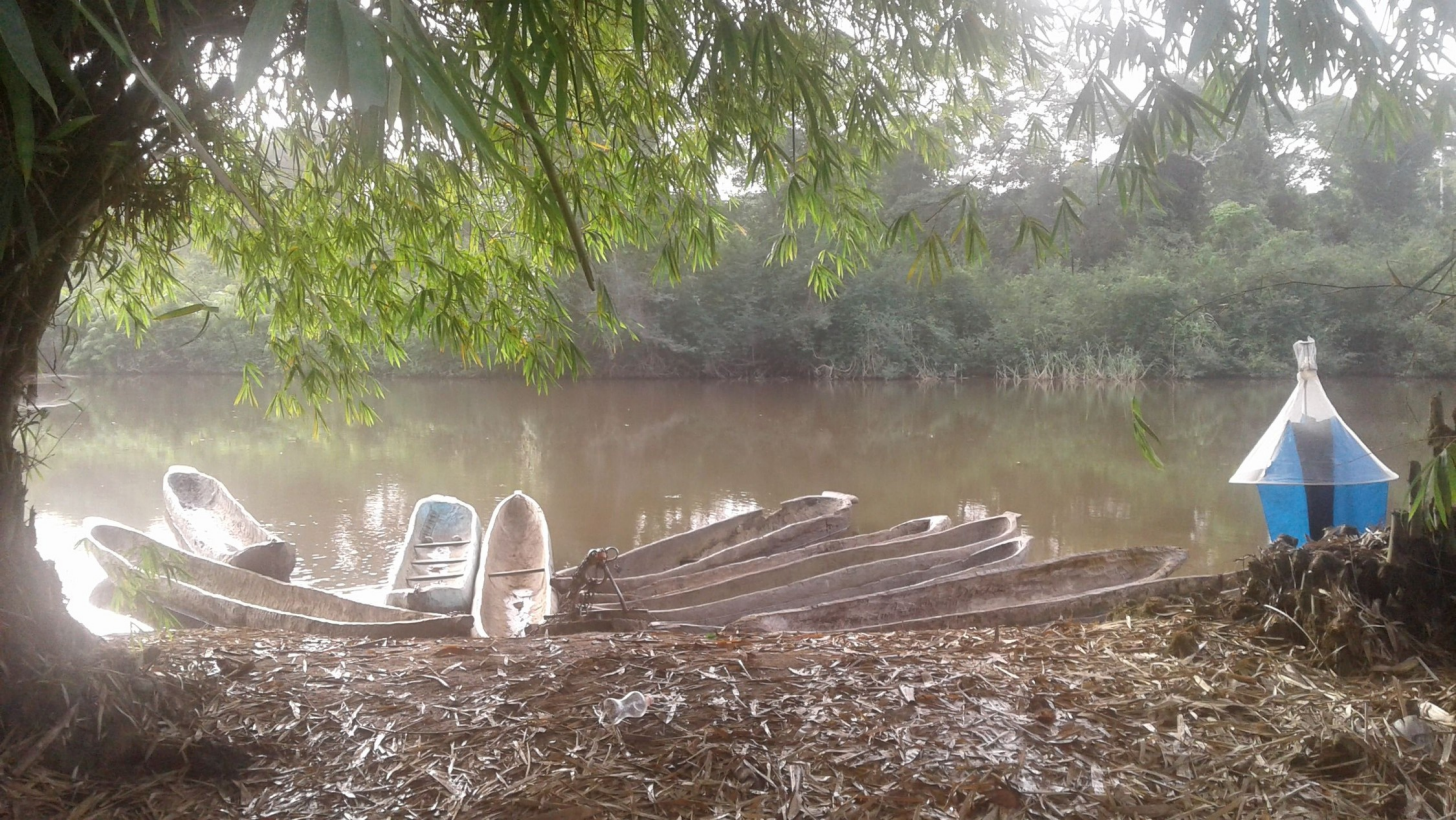
Example of a trap used to catch alive tsetse flies (so without insecticide) on a place frequented by humans.

In g-HAT foci, the first parameter that has to be known is the presence/absence of the vector, its species identification, its geographical repartition in the focus, and its densities. This is done by deploying traps in favourable tsetse habitats in order to catch alive tsetse based on visual and/or olfactory cues (Fig 1). These traps, when used without any insecticide, are used both to assess presence/absence of tsetse as baseline data before any intervention and to monitor tsetse densities to assess the efficacy of control campaigns.

**Fig 2:**
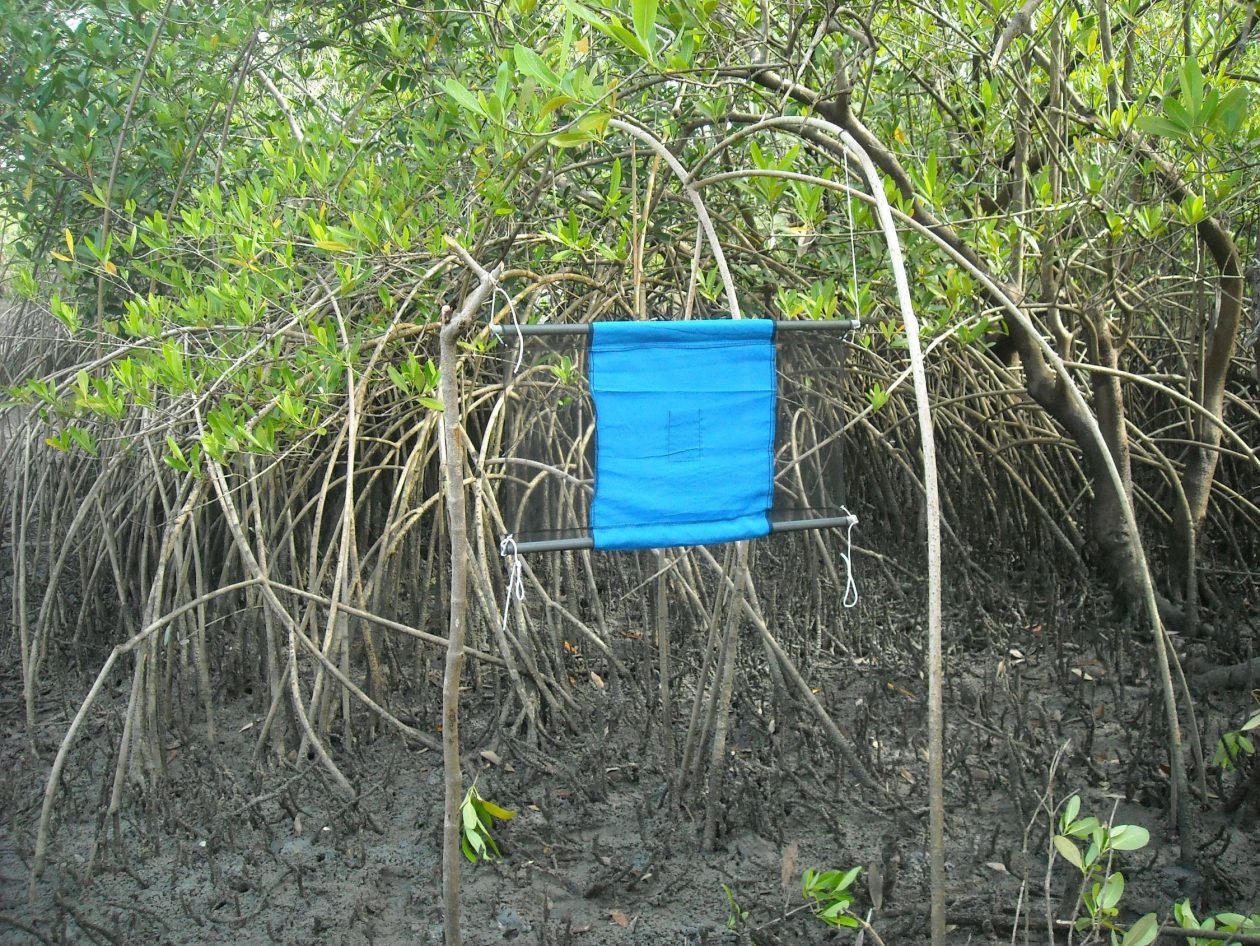
This small piece of cloth impregnated with insecticide (called “tiny target”) is the only prevention mean against g-HAT. When deployed in human–tsetse contact places and used in combination with medical “screen and treat,” it allows breaking transmission cycles.

Nowadays, in active or recently controlled g-HAT foci, vector control is mostly implemented through deployment of insecticide-impregnated small pieces of cloth that are visually attractive to tsetse, so-called “tiny targets” [15,16], see Fig 2. To give an idea of the spatial scale of these control operations, these g-HAT foci are usually in the range of 100 to 1,000 km^2^ each.

In all areas that constitute g-HAT foci where cases have been reported in the past, there are 2 situations: either there are no longer any tsetse or there are tsetse. Let us consider both (see also Table 1).

**Table 1:** Summary of the different situations of g-HAT in endemic countries or health districts and proposed usefulness of entomological criteria to assess g-HAT interruption of transmission.

Type of country/district	Tsetse presence/absence (from literature)	Presence/absence of case	Entomological criteria to assess interrupt of transmission
Country/district with no recently reported HAT case	Tsetse absent	No case	Not relevant
	Tsetse present	No case	Needed
Country/district with reported HAT cases (current or recent)	Tsetse present	No recently reported case	Needed
		No case, but cases recently reported	Mandatory
		Presence of cases	Not relevant because HAT not eliminated

Table 1 summarises the different situations encountered in endemic g-HAT countries or districts regarding presence/absence of cases and of tsetse. In a context where the objective is to assess interruption of transmission, it is proposed to add entomological indicators in g-HAT foci where cases were reported recently. The exact time scale of “recently” (certainly around 5 years) should be subjected to further discussions with experts.

Indeed, there are a number of areas where g-HAT was occurring in the past, but where it has nowadays disappeared due to the disappearance of the tsetse. Such areas include for instance all savannah areas of West Africa that were very prevalent a hundred years ago, and in which g-HAT is no longer found nowadays [17]. In most of these areas, the reason for the disappearance of the tsetse is not a deliberate control action, but is due to global change, that includes mostly human growth and its impact on environment and global warming, both resulting in a destruction of the habitat of the riverine tsetse transmitting trypanosomes to humans. In most of these areas (called “historical foci”) where no case have been reported, and where tsetse have disappeared, we do not see entomological criteria as a priority to assess interruption of transmission. Passive medical detection should remain the main surveillance system. On the other hand, climate change can also result in new areas colonised by tsetse [18] and new risks of HAT transmission, and this should be part of a surveillance system.

In areas where tsetse are still present, as for other vector-borne diseases, any deliberate action aiming at eradicating locally the vector would result in an interruption of transmission of HAT. Eradication has not been implemented in HAT foci in recent time as far as we are aware, but may be interesting to consider since, if successful, this may offer one of the only sustainable ways available of interrupting transmission [19]. Eradication of tsetse has been achieved in a number of places where animal trypanosomosis was occurring, e.g., in Zanzibar [20], or in Botswana [21]. The reason why eradication has not been implemented more often in g-HAT foci are not very clear and may include the difficulty, time, cost, and infrastructures required to achieve eradication.

Nowadays, in most of the current active g-HAT foci (“active” here meaning there is ongoing local transmission), tsetse control campaigns do not aim at eradicating the vector, but rather at protecting people by decreasing tsetse density so that transmission is stopped (e.g., [8]). In areas when cases are no longer being reported but have been reported recently, and where tsetse are still present, our message is that entomological criteria should be used in conjunction with medical data to assess interruption of transmission (see Table 1). Hence, in these foci, the main question to assess interruption of transmission is: do the remaining tsetse carry human-infective trypanosomes? There are currently several tools to answer this question, but these tools all suffer from serious limitations.

## Detection of *T*. *b*. *gambiense* in the tsetse vector: Xenomonitoring

Historically, the usual way of knowing if a tsetse carries trypanosomes was through dissection of the tsetse organs known to harbour trypanosome cyclical development, for *T*. *brucei* the midgut and the salivary glands, followed by parasitological examination [22]. Only trypanosomes found in the salivary gland indicate a *T*. *brucei* mature infection, but it is not possible to know by parasitological examination if the *T*. *brucei* observed is a gambiense or a nonhuman-infective *T*. *brucei*. When trypanosomes are found only in the midgut but not in the salivary glands, the tsetse is infected but it cannot be known if it will be infectious or not for humans. Based on morphology, the trypanosomes found in the midgut can belong either to *T*. *brucei* or to other species (*Trypanosoma congolense* for instance).

The question of the frequency of presence of *T*. *brucei* in the salivary glands of tsetse in natural g-HAT foci has been debated for a long time. It is usually considered that in active foci, there are less than 1% of tsetse with trypanosomes found in the salivary glands. The number of tsetse, which are infected by *T*. *brucei* (all organs), is usually 1% to 10% according to reported studies, depending on the tools used [23].

Molecular tools have been developed, which allow identifying the trypanosomes found in tsetse. However, regarding g-HAT, these molecular tools have not improved the challenge of identifying gambiense in tsetse since the most widely used tools only allow to identify *T*. *brucei*, without knowing if it is a gambiense or not. LAMP has been tried as a cheaper tool than PCR [24], but this did not improve the specificity. Things might have changed when the Tgs-GP primers were designed, because they were described to be specific to *T*. *b*. *gambiense* [25]. However, they have seldom been used as a xenomonitoring tool in HAT foci with the exception of [26] in Uganda, maybe because targeting a single-copy gene may not be sensitive enough.

There is a clear area here for improvement of existing tools, in order to help knowing if the tsetse that are still present in g-HAT foci harbour human-infective trypanosomes. This question is now and more than ever key to assess interruption of transmission.

## Proposal for entomologically based indicators to help assessing interruption of *T*. *b*. *gambiense* transmission

Keeping in mind the need to be able to assess an interruption of transmission, the idea is to propose something around the principle of a TAS-combining information on both the human and vector components applied for g-HAT. We also insist in saying that these only apply to areas that are known to constitute recently active g-HAT foci, i.e., it does not apply to all the area where tsetse occur. In particular as can be viewed in Table 1, there is no need to have an entomological criteria for countries or health districts that have not eliminated the disease, and there is no need to have an entomological criteria for countries or districts that did not report any cases for many years.

But, and this is the strong message of this paper, it is not desirable, and not likely, that a set of parameters based only on the human compartment will be able to confirm this interruption of transmission. There is an absolute need to have entomological parameters for VB-NTDs, and this of course applies to g-HAT.

Hence, the first parameter that needs to be assessed in g-HAT foci recently controlled is the presence/absence of the vector. If the vector is not found, combining absence of new cases in humans and absence of vector provides good data for interruption of transmission. Nonetheless, demonstrating a presence is far easier than demonstrating an absence, including here for tsetse. Hence, it is likely that if the vector is not found, there will be needs for statistical ways of approaching an absence [27] to confirm interruption of transmission.

If the presence of tsetse is confirmed, even more important is to know if these tsetse carry human-infective trypanosomes. For this purpose, xenomonitoring has to be implemented according to one or several of the tools described above, or hopefully to new, better tools that will come from research. Here also the question of the demonstration of absence applies. But considering only the existing tools so far, the presence of a *T*. *brucei gambiense* in any organ of tsetse in a g-HAT focus that has been recently active should certainly be used as a signal to reinforce surveillance. Of course, the same applies when detecting a human case, as recently described in Burkina Faso [28] or modelled in DRC [29].

## Concluding remarks

In the context of elimination of g-HAT, in areas that where transmission was recently ongoing, there is a need to include entomological indicators to assess interruption of transmission as a complement to the medical ones. There is room to improve existing tools of detection of *T*. *b*. *gambiense* in the tsetse vector, or to discover new, better tools, and this should be viewed more than ever as a priority, in order to help creating these indicators as useful steps for countries engaged in this process.
